# Direct measurement of fight or flight behavior in a beetle reveals individual variation and the influence of parasitism

**DOI:** 10.1371/journal.pone.0216387

**Published:** 2019-05-22

**Authors:** Felicia Ebot-Ojong, Elizabeth Jurado, Andrew K. Davis

**Affiliations:** Odum School of Ecology, University of Georgia, Athens, GA, United States of America; Victoria University, AUSTRALIA

## Abstract

How and to what degree an animal deals with potential threats is a fascinating topic that has been well-researched, particularly in insects, though usually not with the impact of parasites in mind. A growing body of work is showing how even benign parasites can affect, positively or negatively, their hosts’ physiological or behavioral reaction to threats. With this in mind we conducted an experiment using horned passalus beetles, *Odontotaenius disjunctus* that were naturally parasitized with a nematode *Chondronema passali*; we subjected beetles to simulated attacks (resembling rival fighting or predator attacks) and from videos of the encounters we quantified a suite of behaviors (antennae movement, aggressive posturing, threat displays, etc.), plus rates of alarm calls (stridulations) which all correspond to the “fight or flight” reaction. We obtained behavioral and parasite data from 140 beetles from two field collections, of which half had been housed in our lab for three weeks in conditions that would be stressful (little cover for burrowing). We observed a wide range of behaviors during the simulated attack procedure, from beetles offering little resistance to those which were extremely aggressive, though most beetles showed a moderate reaction. Alarm calling rates also varied, but surprisingly, these were not correlated with the magnitude of behavioral reactions. Also surprising was that stressful housing did not heighten the physical resistance during attacks, but did elevate alarm calling rate. Importantly, parasitized beetles had significantly reduced physical reactions to attack than those without nematodes (meaning their resistance to the attack was muted). The results concerning parasitism, coupled with prior work in our lab, indicate that the *C*. *passali* nematode depresses the hosts’ acute stress, or fight or flight, reaction (likely from its energetic cost), which may make hosts more susceptible to the very dangers that they are coping with during the stress events.

## Introduction

When animals experience a threat, they are faced with the choice to stay and fight or to flee, and their decision must be carefully weighed (and quickly) or else they may wind up on the losing end of a predator’s jaws or a rival’s kick. The factors that go into this rapid “fight or flight” decision have been well-studied, especially in insects, and include the size of the threat, the previous fighting experience of the individual [1], how much is at stake (i.e. if the individual is defending a brood or territory), and importantly, the physiological status of the animal [2, 3]. In insects, concentrations of the neurotransmitter, octopamine (the chemical cousin to adrenaline) are a key predictor of aggression and propensity to fight, which are both important during physical combat or defense against predators. Octopamine is the insect equivalent to a “stress hormone” since it rises rapidly after any short-term, acute stressor [4–7], and also during longer-term, chronic stressors [8]. The factors that influence contest-winning or fighting behavior have also been well-studied in insects [9–11], and here too, levels of octopamine appear to be important [12]. Collectively, it seems that both the propensity to fight, plus the physical effort exerted during fights are highly dependent on the concentration of octopamine, which is a product of the level of stress the insect is currently experiencing. We note that throughout this fascinating body of work one question has been largely overlooked: what happens in hosts compromised by parasites? Are they more likely to avoid conflict? Or if they choose to fight, can they perform as well during fight or flight situations?

There is reason to expect that parasites could indeed influence this behavior. A small but growing body of evidence across multiple taxonomic groups is showing how parasites, even seemingly benign ones, can affect how, and to what degree, hosts react to acute stress. These effects can either be to heighten the physiological stress response, or to diminish it. For example, amphibians with endo- and ecto-parasites show muted elevations in stress hormones during an acute stress response [13, 14]. Meanwhile, lizards infected with blood parasites showed higher, not lower, stress hormone concentrations during a stress reaction [15]. Rainbow trout (*Oncorhynchus mykiss*) with ectoparasites show higher stress hormone concentrations over time, which leads to muted reactions to acute stress [16]. In contrast, house finches (*Haemorhous mexicanus*) with bacterial infections have higher than normal baseline levels of stress hormones, but they also have heightened stress-induced levels compared to uninfected birds [17]. From these selected examples, two things are clear: acute stress reactions in animals can be positively or negatively affected by a variety of parasites, and, this phenomenon is widespread in nature.

In contrast to their effects on acute stress reactions, the role of parasites during long-term stress situations is well understood. Parasites typically become costly when hosts are faced with prolonged (chronic) stress. One of the most well-known examples of this phenomenon is in soay sheep (*Ovis aries*) in St Kilda, Scotland; individuals infected with intestinal nematodes appear to be unaffected during benign environmental conditions, but during times of prolonged nutritional stress, parasitism is costly and leads to mortality [18]. Similarly, gastrointestinal parasites combined with reduced food availability can lead to reductions in population size of primates [19]. Also, intestinal parasites influence survival of snowshoe hares (*Lepus americanus*) during times of limited food availability [20]. Thus, when hosts are faced with long-term stressful conditions, the (negative) effects of parasitism become magnified.

**Fig 1:**
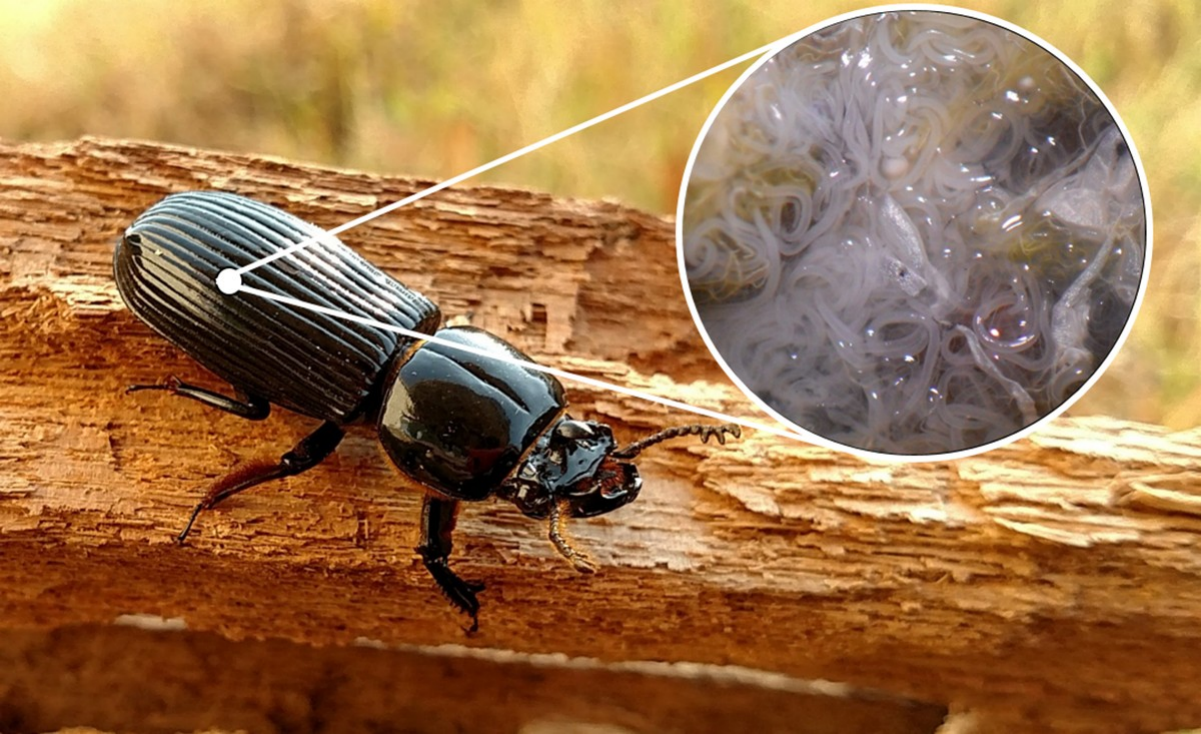
Horned passalus beetle, *Odontotaenius disjunctus*, on a log. This species is common in hardwood forests throughout the eastern United States. Inset image shows its naturally-occurring nematode parasite, *Chondronema passali*, which inhabits the abdominal cavity. Beetle photographed by Stuart Sims, Athens, GA.

In our lab, we have been studying the relationship between stress and parasitism in an insect host, the horned passalus beetle, *Odontotaenius disjunctus* (Fig 1), which is sometimes called the “bess beetle” or “betsy beetle”. This is a medium-sized (1-2g) beetle that ranges throughout the eastern United States and inhabits rotten logs on the forest floor, where it excavates cavities for rearing young. This beetle is host to a number of ecto- and endoparasites [21], though one seems especially relevant, because of its extreme abundance within individuals; *Chondronema passali* is a nematode found in the abdominal cavity of *O*. *disjunctus* (Fig 1) and can number in the thousands in a single beetle [22, 23]. It does not seem to reduce host lifetime fitness, since parasitized individuals are not smaller [24], and appear capable of rearing young (Davis, *pers*. *obs*.). In addition, prevalence of the parasite within local populations is usually close to, or over, 70% [21–23, 25], which speaks to its non-lethal nature. However, like the vertebrate examples above, we have found that it does appear to influence its host during acute stress situations. So far we have examined how this parasite affects various aspects of the stress reaction in horned passalus, including the cardiac response [26], the immune response [27] and the physical performance during stress [9, 25]. All of these efforts have indicated how the parasite appears to (moderately) diminish the hosts’ ability to respond to acute stress situations, presumably because of an energetic cost of the parasite that is only felt when hosts undertake energy-demanding activities. However, we have yet to directly assess the behavior of parasitized beetles during an acute stress scenario, to determine if it (host behavior) is indeed compromised or reduced in any way.

Here we report on an experiment whereby we collected wild passalus beetles (naturally-infected with *C*. *passali*), housed them in our lab for three weeks (in either normal, or stressful conditions), and then subjected them to simulated “attacks” (prodding and pinching with forceps) while filming the encounters. From the videos we numerically quantified how much the beetles resisted, fought back and struggled, using a unique scoring system. We then related their behavioral reactions to their parasite status, to determine if the nematode influences the degree to which beetles respond, in a behavioral sense, to acute stress. Given that our previous work showed that this parasite tends to dampen or slow the host *physiological* response to stress [26, 27], we predicted that parasitized beetles here would show muted *behavioral* reactions to simulated attack. We also expected that beetles housed in chronically-stressful conditions would show heightened acute stress reactions, since chronic stress should elevate levels of octopamine.

Collectively, the results from this study will help to further elucidate the true cost of parasitism in this host, by shining a light on a less-studied period of host biology, but one in which is critical for survival, that is, the fight or flight reaction. Moreover, not only will this help to round out the prior work in this lab on the horned passalus and this parasite, these results should also highlight the value of studying parasites under stressful conditions.

## Materials and methods

Our general approach for this project involved the following steps, which are detailed below: collecting horned passalus beetles and housing them in our lab for 3 weeks, then conducting a behavioral assay of their reactions to simulated attack, and finally assessing beetles for parasitism by the *C*. *passali* nematode. Assessing parasitism status at the end of the project had the advantage of ensuring completely objective assessments of each beetle’s behavior before we knew if it was parasitized.

### Beetle collection and housing

All beetles were collected by hand from forests near the University of Georgia campus (Athens, GA USA) during May and June 2018. Since the University of Georgia owns the properties, no permission was required to collect at these locations. Also, the horned passalus is not an endangered or protected species. We made two collections of beetles for this project, on May 25 (n = 73), and again on June 15 (n = 67); both collections were from different local forests. Beetles were extracted from rotten logs using hatchets and placed in plastic containers for transport back to the lab. There, they were separated and individually housed in 1 liter plastic containers with moist, rotting hardwood. For the purposes of this project, we used two different containers, or housing conditions. We placed half of the beetles (n = 37 for collection 1 and n = 34 for collection 2) in opaque plastic containers (1 liter volume) with abundant, moist hardwood, suitable for burrowing and eating. This is how beetles are typically housed in our lab for research, and we termed this the “normal housing treatment”. The other half (n = 36 and 33) were placed in similarly-sized *clear* (i.e. transparent) plastic containers with only enough wood to eat (not enough to cover the beetle), and these were placed next to a window in the lab. Our intention here was to have these beetles experience a chronically “stressful” environment, where they were both prevented from burrowing and exposed to daylight. Beetles were housed as such for 3 weeks, then we assessed their behavioral reactions to simulated attacks (below).

### Simulating attacks

**Fig 2:**
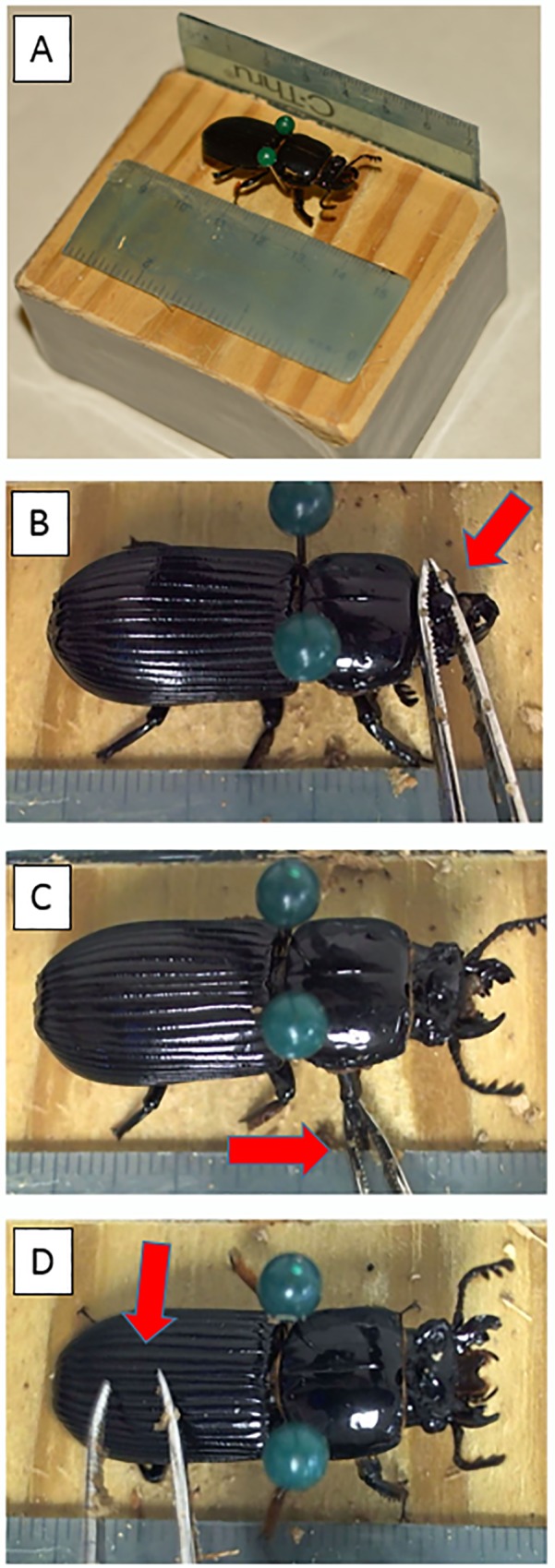
Procedures for recording behavioral reactions to simulated attack. Beetles were positioned on a wooden block and secured in place with two pins placed on either side of its pronotum (A). A video camera was positioned above the block (not shown). The simulated attack lasted 1 minute and consisted of pinching the beetle’s horn (using forceps) for 20 seconds (B), pinching one leg for 20 seconds (C), and tapping on the elytra for 20 seconds (D). Behavioral reactions to these attacks were assessed from the videos of each beetle (see Methods).

The goal of this project was to assess the beetles’ behavioral reactions to simulated attack, such as from a predator or rival beetle. The assessments proceeded as follows: each beetle was positioned on a wooden block under a video camera that was connected to a desktop computer (Fig 2A). The beetle was held in place with two insect pins placed snugly on either side of its pronotum (i.e. not penetrating the beetle, only holding it in place, see Fig 2). Then one of us (F. Ebot-Ojong), using forceps, pinched its single horn and held it pinched for 20 seconds (Fig 2B). Here, we hoped to simulate what would occur during fights between beetles, in which individuals attempt to grab the other with their mandibles [9, 28, 29]. Next, the observer pinched one of the beetle legs for 20 seconds (Fig 2C). This could either simulate a beetle attack or a predator attack. Similar “leg-pinching” has been used to stimulate the stress reactions of other insects (i.e. bees, crickets, [5, 30]). Finally, the observer tapped the elytra of the beetle with forceps for 20 seconds (Fig 2D). While this last stimulus does not simulate any known fighting behavior of this beetle, it may resemble a real-world predator attack, such as a bird poking the beetle, or woodpecker probing the log. Even if this last stimulus does not exactly mirror what would occur in nature, it was effective at eliciting the intended behavioral reaction. In fact, we found that beetles tended to have the strongest reaction during this last phase, as can be seen in Fig 2, by the wide opening of the mandibles, and in the two example videos provided in supplemental files.

### Assessing behavioral reactions

After the simulated attack procedures were completed (but before beetle dissections, below), an observer (F. Ebot-Ojong) watched each beetle video to assess and quantify their behavior during the 1 minute of attack. Note that a third person renamed the videos so that the observer was blind to the housing treatment of each beetle. Plus, since the beetles were dissected only after all behavior data were collected, the observer also did not know the parasite levels of the beetles in the videos. While watching the videos the observer specifically looked for key behaviors typical of passalid and other beetles that reflect aggression, fighting, or escape behavior [9, 28, 29, 31, 32], and which collectively, would show the degree to which each beetle reacted. These include antennal movement (i.e. antennae pressed backward is a sign of submission), mandible position (open mandibles are a sign of aggression and fighting), raising the abdomen (a sign of aggression) and leg movement (submissive beetles tend to curl their legs under them). The observer played back the videos multiple times, making note of each behavior above. We created a scoring system based on these behaviors (Table 1), in which a check was made when one of the behaviors was observed during each of the three phases of the attack (horn pinch, leg pinch, or tapping elytra), and in the end we summed the checks for each beetle to create a single behavioral reaction score to use for analyses. This score ranged from 0 to 26.

**Table 1:** Summary of scoring system used in this experiment to assess the *magnitude* of behavioral reactions observed during simulated attack. Shown is an example score from a beetle with a strong behavioral reaction.

	Attack Phase (20 sec each)		
Beetle Behavior	Horn pinch	Leg Pinch	Tapping Elytra
Antennae extended forward (0 or 1)	1	0	1
Dorsum Elevated (0 or 1)	0	1	1
Mandibles open (0 or 1)	1	1	1
Legs flailing (# legs)	5	6	6
Total for each phase	7	8	9
**Total for this beetle = 24**			

In addition to the physical behaviors we also noted the level of acoustic emissions produced during the attacks, which were readily heard in the videos (see the supplemental videos). Passalid beetles emit a squeaking sound when disturbed or stressed [33], which is generated when they scrape a specialized part of their abdomen against their elytra. This sound can function as a deterrent during predator attacks [34], so it could be considered part of the fight or flight repertoire of these beetles. Thus we counted the number of squeaks emitted during the attacks, which were recorded in the videos. This variable (squeaks per minute) was retained for analyses.

### Assessing nematode parasites

After all behavioral data had been collected, we weighed each beetle, then euthanized and dissected each to determine gender (based on the presence or absence of the male aedeagus) and parasite status. The *C*. *passali* nematode inhabits the abdominal cavity of the beetles and can be readily observed under low-power magnification (see Fig 1). The nematode varies in abundance within beetles, and we scored the parasite burden using a rough scale of 1–3, indicating mild (1–10), moderate (11–100), or heavy (100+) worm burdens [9]. This scoring ended up being unnecessary, as our initial statistical analyses showed minimal effects of various parasite burdens on host behavior (Davis, *unpubl*. *data*). Therefore, we focused only on whether or not the beetles were parasitized with *C*. *passali* nematodes.

### Data analyses

**Fig 3:**
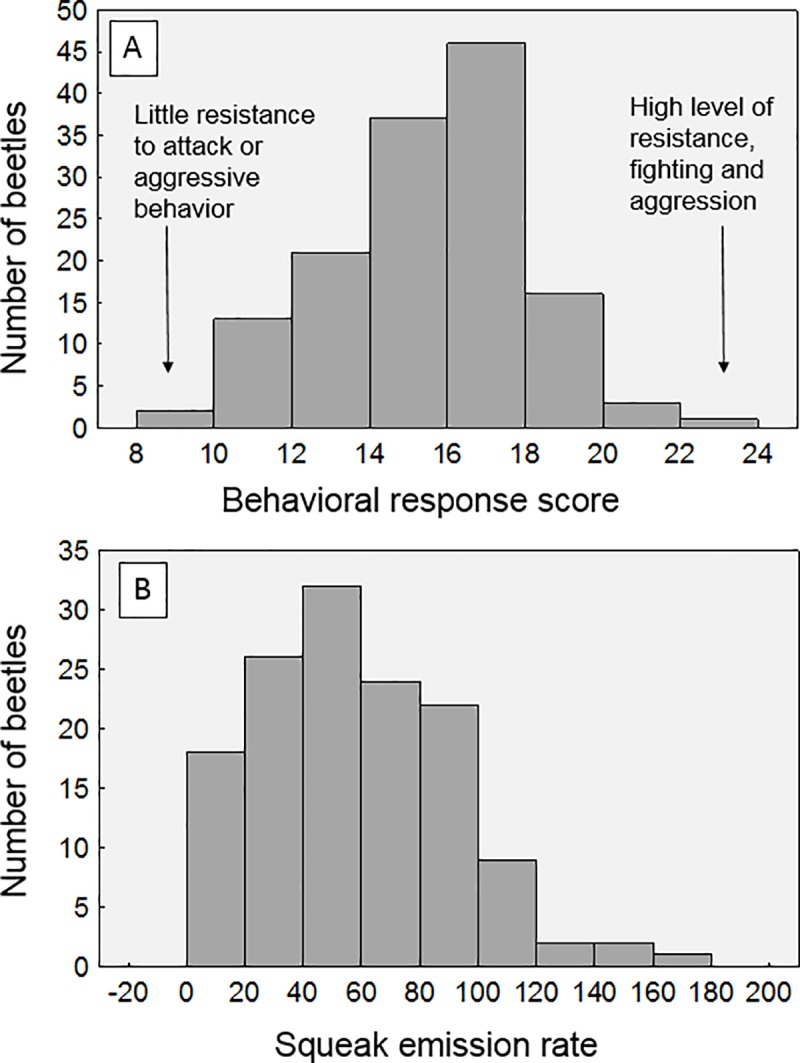
Distribution and range of behavioral responses. Shown are the range of behavioral reaction scores (A) and squeak emission rates (B) during simulated attack for all beetles in this experiment (n = 140).

At the end of this experiment we had obtained behavioral and parasite data from a total of 140 beetles from two field collections. The behavioral data consisted of the numerical behavioral reaction scores and squeaking rates of each beetle (response variables). Both of these variables were approximately normally-distributed (Fig 3). We then used general linear models to determine significant predictors of either response variable. Each model included the following categorical predictors: **collection number** (i.e. first set of beetles or second), **housing treatment** (clear containers with little wood, or opaque containers with ample wood), **parasite status** of the beetle (with or without *C*. *passali* nematodes), and beetle sex. The **beetle mass** was a continuous covariate. Note that we did not include interactions between parasitism and housing treatment, as these were not significant predictors in preliminary models (Davis, *unpubl*. *data*). Statistical analyses were conducted using the Statistica 13.0 software package (TIBCO Software).

## Results

### General observations

The full dataset for this project is available online in the supplemental files for this paper. We observed a range of behaviors during the simulated attack procedure, from beetles offering little resistance to those which were extremely aggressive. This range is visible in Fig 3A, which shows the distribution of the numerical scores per beetle. Beetles that had high scores were those that flailed all their legs vigorously, raised their abdomen, and showed wide-open mandibles throughout the attack. Conversely, some beetles showed little resistance when their horn or legs were pinched, and/or showed little leg movement. These contrasting behaviors are evident in the two supplemental videos provided, which show beetles at both ends of this spectrum. Similarly, the rate of acoustic emission during attack also varied between individuals (Fig 3B). We noted that the rate of acoustic emissions was not correlated with the behavioral scores, based on Pearson correlation test (n = 136, r = -0.0402, p = 0.642).

### Predictors of behavioral reactions

**Fig 4:**
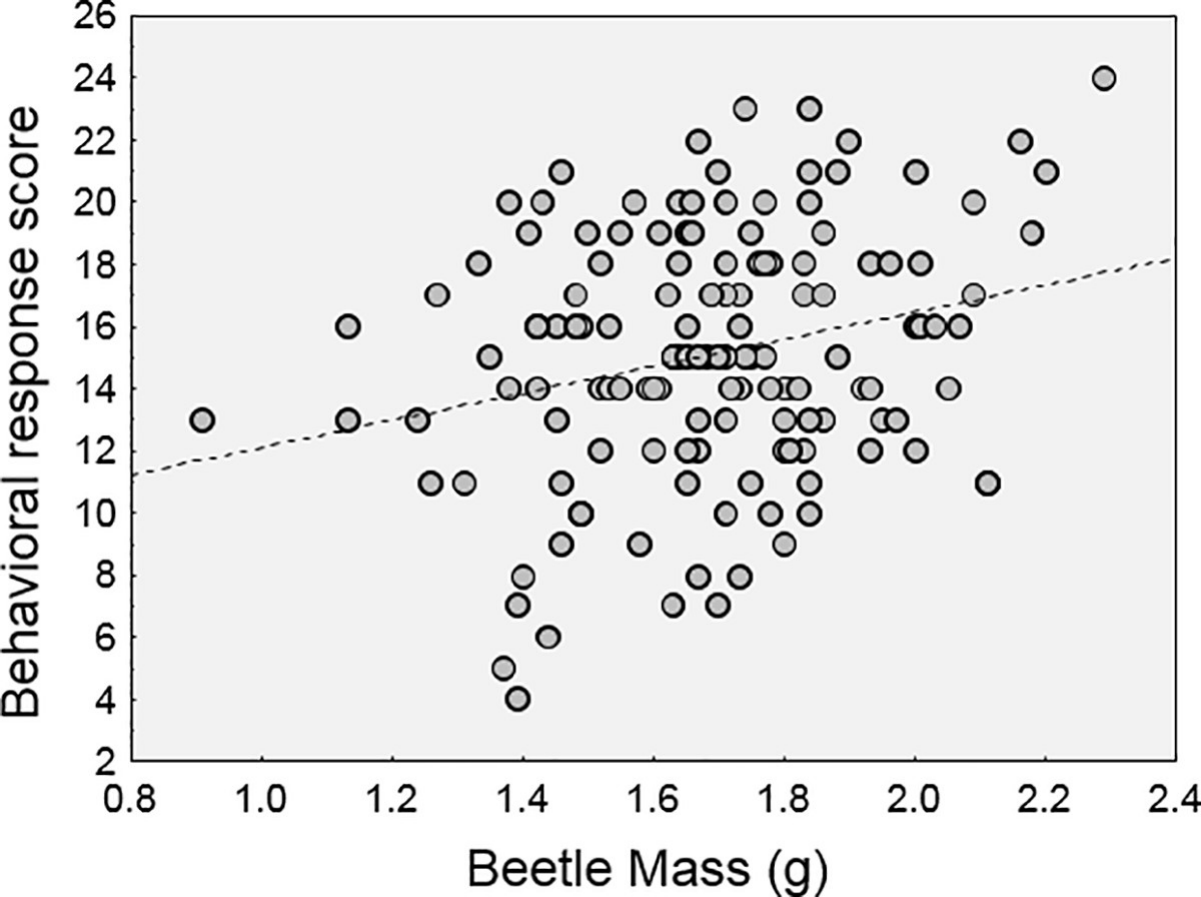
Relationship between beetle size (body mass) and behavioral response. There was a positive relationship between body mass and reactions to simulated attack.

**Fig 5:**
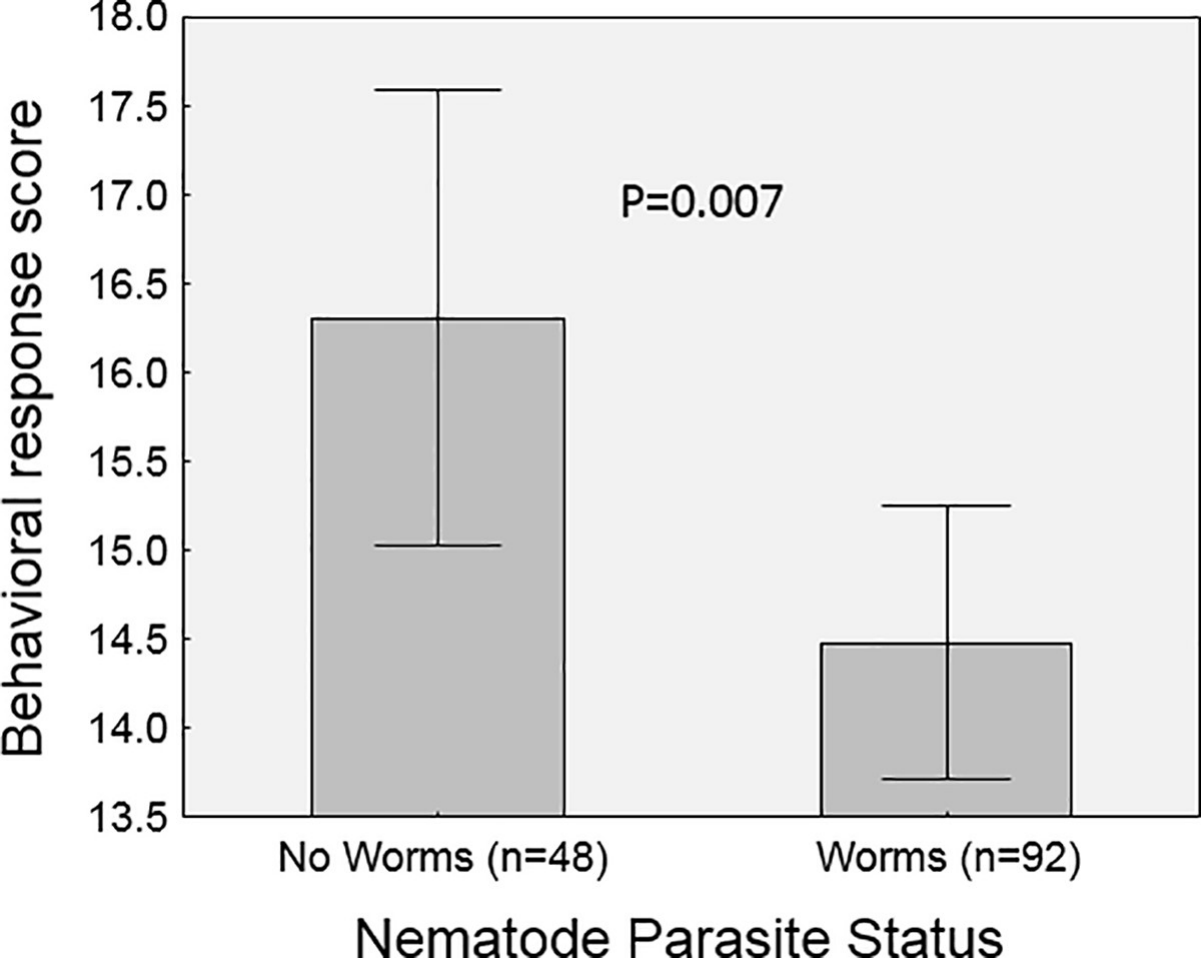
Effect of nematode parasites (C. passali) on behavioral responses. Shown are mean behavioral response scores (with 95% CI) of parasitized and non-parasitized beetles during simulated attack. Parasitized beetles had significantly lower reactions to simulated predator attack than beetles without parasites.

In the model that examined predictors of the reaction scores, there were two significant main effects (Table 2). The reaction scores varied with the size of the beetle (p = 0.0125). This effect was positive (Fig 4); larger beetles tended to show greater resistance and aggression during the simulated attacks. The presence or absence of *C*. *passali* nematode parasites also affected the degree to which beetles reacted to the attacks (p = 0.0230). Here, parasitized beetles had lower average scores than those without nematodes (Fig 5). Based on these means, we estimate this difference to be approximately 11%.

**Table 2:** Summary of general linear model that examined predictors of behavioral response scores (during simulated attack) in horned passalus beetles in this experiment. An interaction term between parasitism and housing treatment was not significant in a preliminary model (Davis, *unpubl*. *data*).

Predictor	Degrees of freedom	Mean Squares	F statistic	P value
**Beetle Mass**	1	96.67	6.4	0.0125
**Beetle Collection Number (1 or 2)**	1	16.90	1.12	0.2917
**Housing Treatment**	1	17.75	1.17	0.2798
**Parasite Status (infected, uninfected)**	1	79.76	5.29	0.0230
**Beetle sex**	1	0.63	0.04	0.8376
**Error**	133	15.08		

## Predictors of acoustic emissions

**Fig 6:**
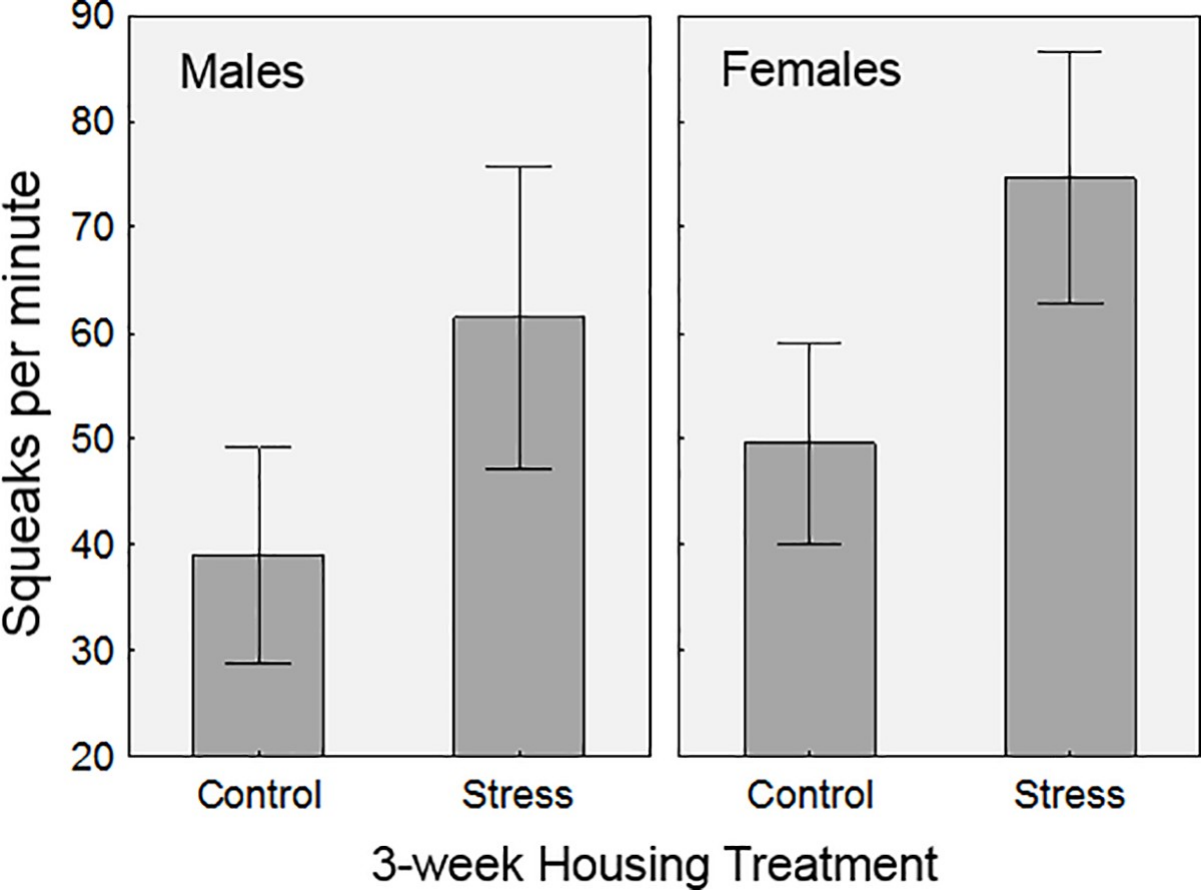
Average acoustic emission rates (during simulated attack) in relation to housing. Shown are frequencies of squeaks of male and female beetles that were housed for 3 weeks in two different conditions: normal housing (opaque container with ample wood for burying), and stressful housing (clear container, beetles prevented from burying). Whiskers represent 95% confidence intervals.

The model describing which predictors affected beetle squeaking rate (Table 3) did not resemble the aforementioned model of behavioral reaction scores. Here, beetle size did not affect acoustic emission rates (p = 0.1630), nor did nematode status (p = 0.7216). And in this model, the housing treatment did matter (p = 0.0001). Finally, there was a significant difference between sexes in the acoustic emission rate (p = 0.0332). Graphing the emission rate of male and female beetles in the two different housing treatments indicates those in the stressful conditions squeaked more than those in the normal containers (Fig 6). Also evident is that females tended to squeak more frequently than males.

**Table 3:** Summary of general linear model that examined predictors of acoustic emission rates (during simulated attack) in horned passalus beetles in this experiment. An interaction term between parasitism and housing treatment was not significant in a preliminary model (Davis, *unpubl*. *data*).

Predictor	Degrees of Freedom	Mean Squares	F statistic	P value
**Beetle Mass**	1	2237.39	1.97	0.1630
**Beetle Collection Number (1 or 2)**	1	65.65	0.05	0.8104
**Housing Treatment**	1	17335.63	15.24	0.0001
**Parasite Status (infected uninfected)**	1	144.97	0.13	0.7216
**Beetle sex**	1	5269.84	4.63	0.0332
**Error**	130	1137.00		

## Discussion

The concept of the “fight or flight” stress response was first presented in 1929 [35] as a way to describe the collective suite of bodily reactions to stressful stimuli in humans. This phrase is still used today to refer to the suite of physiological and behavioral reactions in humans or human models [36] and even insects [3, 5], though it inherently conjures an image of a binomial response, where animals can either choose to stay and fight or flee, i.e. one or the other [3]. Interestingly, this is not what we observed throughout this experiment, though perhaps this was because our setup did not allow the beetles to choose flight (they were held in place). Even still, we might have expected to see individuals fall into two overall groups, i.e. those that showed heightened resistance and vigorous fighting, versus those that offered little resistance. In contrast, our observations suggest that this reaction (at least in the horned passalus) is more of a spectrum of behaviors (Fig 3A), rather than a choice of two opposing options. In fact, based on the numerical scoring system we used to quantify reactions to attack, we noted that the majority of individuals showed a moderate reaction somewhere between the two extremes on either end (Fig 3A). Other insect investigators have reached similar conclusions on this topic–that the stress response is more of a continuum, and not an all or nothing event [37].

When designing this experiment, we focused on two components of behavior that we felt would collectively represent the fight or flight reaction in passalid beetles–their *physical* reactions, plus their *acoustic* emissions. Interestingly, we found that these two behaviors are not influenced similarly by external or internal factors; the two statistical models (Tables 2 and 3) revealed each variable was affected by different predictors. Moreover, the two behaviors are not correlated among individuals. The lack of correlation between behavioral reactions and acoustic emission implies a beetle could mount a large physical response to an attack, but emit little to no squeaking during the attack, and conversely some beetles could squeak a lot, but yet make few physical movements during the attack. It may be that these behaviors are separated because of the amount of energy required to perform each; perhaps the physical reactions require more energy while squeaking requires little. Indeed, the physical reactions involve moving and flailing major body parts (legs, mandibles, abdomen) sometimes all at once, while the acoustic emissions are derived by merely rubbing a small part of the abdomen against the underside of their wings [34]. That these behaviors may require different amounts of energy may also be the reason also for why nematode parasites affected the physical but not the acoustic reactions (below).

Another reason to believe the physical reactions to attack are a function of energy availability is the effect of beetle size we observed. Larger beetles tended to (physically) react more strongly to attack (Fig 4). In most beetle species, large body size is usually a key predictor of fight success [38, 39], and this is usually attributed to larger beetles having larger muscles. While it is true that larger beetles are physically stronger, even in horned passalus [10, 24, 40], our results suggest that larger beetles also “react” more vigorously (to attacks) in a behavioral sense, i.e. they put up more of a fight. This implies that they committed more energy to the fight, which in turn implies they had more energy to do so. This idea makes intuitive sense, although we admit have not yet explicitly examined energy availability in parasitized beetles.

We designed this experiment largely to answer the question of how parasites influence the fight or flight reaction of their host. Our study showed that internal *Chondronema passali* nematode parasites can indeed reduce certain components of their host’s reaction to attack: the level of physical resistance, or the effort spent fighting back. This effect was small (~11%), but statistically significant. With the scoring system we used, it is difficult to pinpoint exactly which element of the reaction was most influenced (i.e. legs, vs mandibles, etc.). We note though that this result is consistent with other studies from our lab which have focused on this host-parasite system, which collectively show that the effect of this parasite appears to be felt most strongly during energy-intensive situations. For example, parasitized passalus beetles are slightly less successful in fights against other beetles, and their fights are less energetic [9]. During acute stress situations, parasitized beetles show muted physiological reactions, including the cardiac response [26] and the immune system response [27]. In addition, the beetles’ physical strength has been directly tested also in both passive and in stressed situations; when beetles were allowed to passively pull a weight in a tunnel there was no effect of nematode parasites [24], but when made to lift a weight while being prodded (stressed), there was a negative effect of parasitism [25]. The current study adds to this growing literature and emphasizes how the effects of parasites to their host may be felt most during times of acute stress.

Finally, as part of this experiment, we were also interested in determining if chronic stress (stressful housing) changes the way beetles react to the acute stressor (simulated attack). Given that long-term stress in invertebrates tends to lead to elevations in octopamine, the insect equivalent to adrenaline [3, 8], and that higher octopamine is linked with increased aggression [2, 41], we expected to observe heightened behavioral reactions in the stressed-housed beetles. We observed mixed results depending on which aspect of the beetle behavior we examined. Chronic stress did not affect the physical response to attack, but it did alter the alarm calling rate of beetles; beetles housed in stressful conditions “squeaked” more often during simulated attack (Fig 6). Passalid beetles squeak when handled and this squeaking has been shown to deter predators [34]. This suggests that this acoustic behavior reflects the current physiological state of the beetles. From a research standpoint, this information could be useful in assessing levels of stress in collections of beetles from locations that are suspected of being “stressful”, such as near noisy roadways [42], i.e. by comparing “squeak rates” (in response to handling) between disturbed and undisturbed sites.

## Conclusions

We conducted an experiment assessing behavioral reactions to simulated attack in beetles that were naturally infected with nematodes, and that were held in stressful environments to determine the effect of either condition on the overall fight or flight reaction. We derived a novel scoring system for determining the magnitude of the reaction, and this showed more of a continuum of reactions rather than a binomial response. The degree of physical resistance during attack was slightly, but significantly, lower in parasitized beetles, consistent with earlier work showing how parasites reduce the stress response. Chronic stress did not lead to heightened physical reactions, but it did cause greater distress calls during simulated attacks. We conclude that parasitism by *Chondronema passali* nematodes imposes a cost to the beetles’ acute stress reaction presumably because of a mild energy drain by the parasite, and the energy demand during the acute stress reaction.

## Supporting information

S1 File Beetle with low score.wmv. Video showing an example beetle which had a low behavioral reaction to simulated attack.
(WMV)

S2 File Beetle with high score.wmv. Video showing an example beetle which had a high behavioral reaction to simulated attack.
(WMV)

S3 File Copy of All Data for Supplemental File—Final. All raw data used in the analyses of the project.
(XLSX)
